# Effectiveness of the spot-on combination of moxidectin and imidacloprid (Advocate®) in the treatment of ocular thelaziosis by *Thelazia callipaeda* in naturally infected cats

**DOI:** 10.1186/s13071-018-3262-1

**Published:** 2019-01-11

**Authors:** Domenico Otranto, Fabrizio Solari Basano, Marco Pombi, Gioia Capelli, Roberto Nazzari, Luigi Falsone, Gabriele Petry, Matthias Günter Pollmeier, Riccardo Paolo Lia

**Affiliations:** 1 Dipartimento di Medicina Veterinaria, Università degli Studi di Bari, 70010 Valenzano, Italy; 2 Arcoblu s.r.l., Via Alessandro Milesi 5, 20133 Milano, Italy; 3 Dipartimento di Sanità Pubblica e Malattie Infettive, Sapienza Università di Roma, 00185 Roma, Italy; 4 Istituto Zooprofilattico Sperimentale delle Venezie, 35020 Legnaro, Italy; 5 Bayer Animal Health GmbH, Leverkusen, Germany

**Keywords:** Advocate, Eyeworm, Zoonosis, Moxidectin, Parasite, *Thelazia callipaeda*, Treatment

## Abstract

**Background:**

The present study evaluated the therapeutic effectiveness of moxidectin 1.0% (w/v) and imidacloprid 10% (w/v) (Advocate® spot-on solution for cats, Bayer Animal Health) against natural infections with the eyeworm *Thelazia callipaeda* in cats. This study was conducted as a GCP, negative-controlled, blinded and randomised field study in privately owned cats living in an area in southern Italy where *T. callipaeda* is enzootic.

**Methods:**

The study was carried out in 30 cats (19 females and 11 males, aged from 8 months to 5 years, weighing 1.2–5.2 kg) of different breeds, naturally infected by *T. callipaeda.* At study inclusion (Day 0), animals were physically examined and the infection level was assessed by examination of both eyes for clinical score and live adult *T. callipaeda* count. Each cat was weighed and randomly assigned to one of the treatment groups (G1: Advocate, G2: untreated control). Clinical assessments and *T. callipaeda* adult counts were performed on Day 14. At the study completion visit on Day 28, clinical assessments and counts of *T. callipaeda* adults and larvae were performed. All cats were daily observed by their owners and general health conditions were recorded during the entire period of the study.

**Results:**

The primary effectiveness variable was the percentage of animals in G1 group (Advocate) showing a complete elimination (parasitological cure) of adult eye worms at Day 14 and Day 28 . The effectiveness of the treatment in the G1 group was 93.3 and 100% at Day 14 and Day 28 , respectively, when compared to group G2. Total worm count reduction from both eyes for Advocate was 96.3% on Day 14 and 100% on Day 28. Clinical data were confirmed by the examination of conjunctival pouch flushing. An overall reduction in the number of cats with lacrimation and conjunctivitis was observed following treatment despite the fact that in a few cats treated with Advocate clinical signs persisted due to the chronic nature of the disease.

**Conclusions:**

Based on the results of the present trial, a single dose of Advocate was found to be safe and highly effective in the treatment of natural *T. callipaeda* infection in cats.

## Background

*Thelazia callipaeda* is a spiruroid nematode residing in the conjunctival pouches of dogs, cats, wild carnivores and humans. Since its first description in dogs from northern Italy [1], reports of thelaziosis have increased showing a wide distribution throughout European countries and its capability to provoke ocular manifestations such as conjunctivitis, keratitis, corneal opacity and ulcers [2–4] due to the mechanical action of the adult parasite on the conjunctival and corneal epithelium. Due to its original distribution in East Asian countries, this worm used to be regarded as the “oriental eye-worm”, but after more recent descriptions in animals and humans throughout European countries such as Italy [5], Spain [6, 7], Croatia [8], Greece [9], Serbia [10], Bulgaria and Hungary [11], this term is no longer appropriate. The increase of infections in both animals and humans is related to the presence of the *T. callipaeda* vector, *Phortica variegata*, a drosophilid of the subfamily Steganinae which, in Europe, acts as intermediate host of the eyeworm [12, 13].

*Phortica variegata* live in forested and meadow areas where several competent wild hosts (e.g. red foxes, wolves, beech martens and wild cats [14]) perpetuate the infection, being reservoirs of the infection to dogs and cats [15]. Among available treatment procedures, physical removal of nematodes from conjunctival pouches consisting in a saline rinse (effective for adult and immature nematodes) and mechanical adult nematode collection by fine forceps or swabs has been described [16]. For canine thelaziosis, many treatments have been proposed, including topic instillation of organophosphates [17] or moxidectin [18]; however, these had major local side effects.

In 2016, Otranto et al. [19] published results on the effectiveness of moxidectin 2.5% w/v and imidacloprid 10% w/v (Advocate® spot-on solution for dogs, Bayer Animal Health) in thelaziosis showing a high effectiveness: a single application of this drug was sufficient to safely eliminate 100% of nematodes in infected dogs within seven days after a single administration. Moreover, the latter formulation acts systemically after topical application and consequently ocular irritation as local side effect while treating eye worm infections will not occur. In cats, the administration of milbemycin oxime at 2 mg/kg was demonstrated to have a high effectiveness in the treatment of *T. callipaeda* infections [20], but no registered products are currently available for the treatment of thelaziosis in this species.

The aim of this study was to evaluate the effectiveness of moxidectin 1.0% w/v and imidacloprid 10% w/v (Advocate spot-on solution for cats, Bayer Animal Health) against natural *T. callipaeda* infection in cats.

## Results

At baseline all cats were infected by *T. callipaeda*. The average number of adult *T. callipaeda* specimens observed in G1 and G2 was 2.13 and 1.87, respectively. The number of parasites and the number of cats with clinical manifestations (lacrimation, conjunctivitis and ocular discharge; Table 1) in each eye was homogenous among groups (*F*_(1,28)_ = 0.767, *P* = 0.389 and Fisher’s exact test, *P* = 0.500, for the number of parasites and for lacrimation, conjunctivitis and ocular discharge, respectively). Keratitis and ulcers were absent in all the cats. The number and percentage of cats positive for *T. callipaeda* infection at each study day are reported in Table 2. Effectiveness in G1 (Advocate) was 93.3% on Day 14 and 100% on Day 28. The total number and mean of live *T. callipaeda* adults retrieved and counted on both eyes is reported in Table 2. The reduction in the number of worms counted in both eyes was 96.3% on Day 14 and 100% on Day 28 for the treated group. A natural mild decrease was observed for the untreated group showing a worm reduction of 3.6% on Day 14 and 7.1% on Day 28. The comparison of worm reduction between groups showed a significant difference at all post-treatment visits when tested by ANOVA (*F*_(1,28)_ = 67.600, *P* < 0.0001). Among ocular signs associated with *T. callipaeda* presence, only lacrimation, conjunctivitis and discharge were recorded in the included cats; details of observed symptoms are reported in Table 1. None of the cats exhibited keratitis and ulcers except one cat in the untreated group that showed both symptoms at the study closure visit (Day 28). A significant difference between treated and control groups were observed for lacrimation on Day 28 (Fisher’s exact test, *P* = 0.028) and for conjunctivitis on Day 28 (Fisher’s exact test, *P* = 0.020). No treatment related adverse effects were recorded.

**Table 1:** Number (and percentage) of cats showing clinical manifestations associated to *T. callipaeda* infection at different time points of the study

Symptom	D0	D14	D28
Lacrimation			
G1	10 (66.6)	5 (33.3)	5 (33.3)^a^
G2	9 (60.0)	10 (66.6)	11 (73.3)^a^
Conjunctivitis			
G1	3 (20.0)	1 (6.7)	2 (13.3)^b^
G2	4 (26.7)	3 (20.0)	8 (53.3)^b^
Discharge			
G1	1 (6.7)	0 (0)	0 (0)
G2	0 (0)	0 (0)	2 (13.3)
Keratitis			
G1	0 (0)	0 (0)	0 (0)
G2	0 (0)	0 (0)	1 (6.7)
Ulcers			
G1	0 (0)	0 (0)	0 (0)
G2	0 (0)	0 (0)	1 (6.7)

*Abbreviations*: *G1* treated at D0 with Advocate, *G2* untreated control, *D* study day

^a^*P* = 0.028

^b^*P* = 0.020

**Table 2:** Number (and percentage) of cats positive for adult *Thelazia callipaeda*, count and mean of detected adult nematode, worm reduction and efficacy of treatment (G1) during the study

Group	Response variable	D0	D14	D28
G1	Positive cats, *n* (%)	15 (100)	1 (6.7)	0
	Adult count (mean)	32 (2.13)	1 (0.07)	0
	Reduction %		96.3	100
G2	Positive cats, *n* (%)	15 (100)	15 (100)	15 (100)
	Adult count (mean)	28 (1.82)	27 (1.80)	26 (1.73)
	Reduction %		3.6	7.1
	Efficacy %		93.3	100

*Abbreviations*: G1, treated at D0 with Advocate; G2, untreated control; D, study day

## Discussion

A single spot on application of Advocate, containing moxidectin 1.0% (w/v) and imidacloprid 10% (w/v), was highly effective in the treatment of feline thelaziosis. Only one cat out of 15 treated was found positive two weeks after treatment and no worms were detected four weeks after treatment administration. No side effects were observed during the whole study period. As was previously demonstrated for dog patients [21], this commercial formulation showed to be safe and effective for the treatment of *T. callipaeda* infection in feline patients. In this study a low prevalence of ocular clinical signs was observed, confirming that the disease has a high variability in the clinical presentation and that the occurrence of subclinical asymptomatic infections is common. Even if some clinical signs showed a significant reduction (lacrimation and conjunctivitis) in treated cats, the observation of the persistency of clinical signs in a few treated animals, also following parasitological cure is due to the nature of the disease that frequently has a chronic course. Concerning the “One Health” approach, as reported elsewhere [15], cases of human thelaziosis are reported in areas where the infection is highly prevalent in the animal population. Cats are hosts that can often reach high densities in peridomestic habitats; therefore, a safe and effective drug being approved for the treatment of feline thelaziosis represents an important asset in *T. callipaeda* control.

## Conclusions

The results of the present study demonstrate that Advocate is safe and highly effective in the treatment of *T. callipaeda* infection in cats after single administration of the recommended dose rate.

## Methods

**Fig. 1:**
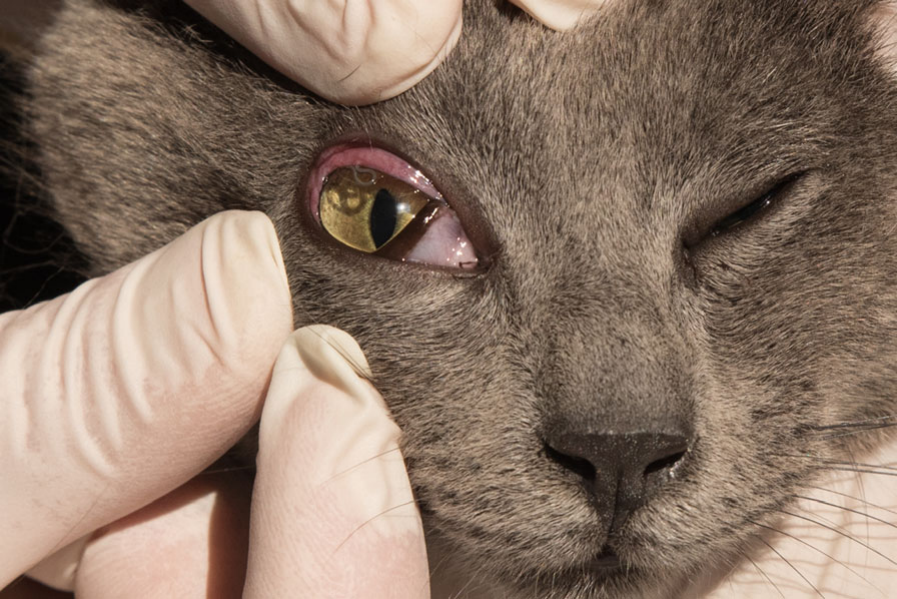
*Thelazia callipaeda* nematodes on the conjunctiva of a cat from the study

The study was conducted as a Good Clinical Practice (GCP), negative controlled, blinded and randomized field study in privately owned cats living in a *T. callipaeda* enzootic area of the Basilicata region (southern Italy), following approval by the Italian Ministry of Health (DGSAF- 0028002-06/12/2016-DGSAF-MDS-P). Study animals were located in areas enzootic for thelaziosis where the presence of the vector and of the diseases in dogs and cats was already proven [20, 22]. A sample size of 30 cats, 15 per group, was estimated to detect a difference between proportions (i.e. % of non-infected cats) in treated animals *versus* untreated, assuming a prevalence of infected cats in the untreated group of 73.3% (Motta et al. [20]) and a confidence level of 95% [software nQuery+nTerim 3.0 (StatSols, Statistical Solutions© Ltd. 2014). Thirty cats (19 females and 11 males) aged from 8 months to 5 years, of different breeds, in good health conditions and with at least one live adult *T. callipaeda* nematode in one eye (Fig. 1) were included in the study following the collection of the owner informed consent form. Details are provided in Table 3.

**Table 3:** Description of the characteristics of the cats included in the study. Ranges for age and weight are reported in parentheses

	G1 (*n* = 15)	G2 (*n* = 15)	Total (*n* = 30)
Age (months)	20.7 (8–60)	23.5 (9–48)	22.1 (8–60)
Sex (no. of cats)^a^	12/3	7/8	19/11
Weight (kg)	2.7 (1.2–5.2)	2.8 (1.2–4.3)	2.8 (1.2–5.2)
Hair length (no. of cats)^b^	0/14/1	0/12/3	0/26/4

*Abbreviations*: *G1* treated at D0 with Advocate, *G2* untreated control

^a^Female/Male

^b^Long/Medium/Short

At inclusion (D0) animals were physically examined, weighed and inspected at both eyes, including a thorough examination underneath the third eyelid to retrieve and count live adult of *T. callipaeda.* Clinical manifestations suggestive of eye worm infection such as lacrimation, conjunctivitis, ocular discharge, keratitis, and ulcers, were recorded and classified as absent, mild, moderate or severe.

Allocation to study groups (G1: Advocate, G2: untreated) was done in accordance with a random treatment allocation plan. In order to avoid bias due to the contact between treated and untreated cats, animals of the same household were allocated to the same study group. Advocate was administered on the basis of the body weight, following the instructions reported on the label of the commercial packaging to provide a minimum spot-on dose of 1.0 mg/kg moxidectin and 10 mg/kg imidacloprid.

On Day 14 and Day 28 (study closure) cats were physically examined and the infection was evaluated by inspection of both eyes, including conjunctival pouches and third eyelids for live adult *T. callipaeda* count and clinical scores.

At Day 28, the final visit, the conjunctival pouch of both eyes of each animal was flushed twice with 2.5 ml of 0.9% saline solution to collect samples for the evaluation of the presence of *T. callipaeda* adults and larvae. The instilled liquid was recollected by placing a Petri dish right under the eye and transferred into a tube that was centrifuged for 5 min at 700× *g*. The supernatant was aspirated and the sediment (1 ml solution) assessed by microscopic examination (40×) for the presence of parasite stages. Nematodes were counted and morphologically identified according to Skrjiabin et al. [23] and Otranto et al. [24].

From Day 0 to Day 28 all cats were observed daily by their owners to assess and record potential abnormalities of the general health and, in case of their occurrence, the veterinarian was responsible for examining the cat and recording results of the clinical examination.

The primary variable for the effectiveness evaluation was the number of cats showing a complete elimination of adult eye worms on Day 14 and Day 28 by comparison of G1 (treated cats) and G2 (untreated cats). As secondary descriptive parameters, worm count, presence and/or severity of ocular clinical signs were calculated and compared between the two groups on Day 14 and Day 28. Effectiveness (%) in the treatment of *T. callipaeda* infection was calculated for each time point using the following formula:

$$ \mathrm{Effectiveness}\ \left(\%\right)=\left(\mathrm{Positive}\ \mathrm{animals}\ \mathrm{in}\ \mathrm{the}\ \mathrm{untreated}\ \mathrm{group}-\mathrm{Positive}\ \mathrm{animals}\ \mathrm{in}\ \mathrm{the}\ \mathrm{treated}\ \mathrm{group}\right)/\mathrm{Positive}\ \mathrm{animals}\ \mathrm{in}\ \mathrm{the}\ \mathrm{untreated}\ \mathrm{group}\times 100 $$

Effectiveness was claimed if a significant difference between groups G1 and G2 was demonstrated by Fisher’s exact test calculated on contingency tables for parasitological cure with 5% significance level of probability of rejecting the null hypothesis.

Worm count and ocular clinical signs reductions in the treated group were compared at time points (t) Day 14 and Day 28 with the untreated group as follows:

$$ \%\mathrm{reduction}\ \left[\mathrm{t}\right]=\left({\mathrm{C}}_{\mathrm{t}0}-{\mathrm{C}}_{\mathrm{t}}\right)/{\mathrm{C}}_{\mathrm{t}0}\Big)\times 100, $$

where C_t0_ was the baseline count before treatment and C_t_ was the count at time t after treatment for each secondary variable under investigation. The significance of the worm count reduction in treated cats was analyzed by ANOVA with standard statistical assumption. Statistical analysis was planned and conducted in compliance with current guidelines [25]. Statistical calculations and randomization were performed with: SPSS® statistical package for Windows, v.23.0 and nQuery + nTerim 3.0 (StatSols).

## Abbreviations

GCP: Good Clinical Practice
G1: Study group of cats treated with imidacloprid 10% and moxidectin 1.0% spot-on
G2: Study group of untreated cats
Day 0: Day of inclusion of selected cats
Day 14: 14th day of the study
Day 28: 28th day of the study, closure
